# GRam stain-guided Antibiotics ChoicE for Ventilator-Associated Pneumonia (GRACE-VAP) trial: rationale and study protocol for a randomised controlled trial

**DOI:** 10.1186/s13063-018-2971-2

**Published:** 2018-11-08

**Authors:** Jumpei Yoshimura, Kazuma Yamakawa, Takahiro Kinoshita, Yoshinori Ohta, Takeshi Morimoto

**Affiliations:** 1 Division of Trauma and Surgical Critical Care, Osaka General Medical Center, 3-1-56 Bandai-Higashi, Sumiyoshi, Osaka, 558-8558 Japan; 2 Division of General Medicine, Department of Internal Medicine, Hyogo College of Medicine, 1-1 Mukogawa, Nishinomiya, Hyogo 663-8501 Japan; 3 Department of Clinical Epidemiology, Hyogo College of Medicine, 1-1 Mukogawa, Nishinomiya, Hyogo 663-8501 Japan

**Keywords:** VAP, Gram staining, Antimicrobial therapy, Empirical therapy, Nosocomial infection, Mechanical ventilation, ICU, MDR, RCT

## Abstract

**Background:**

Optimising the use of antibiotic agents is a pressing challenge to overcoming the rapid emergence and spread of multidrug-resistant pathogens in intensive care units (ICUs). Although Gram staining may possibly provide immediate information for predicting pathogenic bacteria, Gram stain-guided initial antibiotic treatment is not well established in the ICU setting. We planned the GRam stain-guided Antibiotics ChoicE for Ventilator-Associated Pneumonia (GRACE-VAP) trial to investigate whether Gram staining can safely restrict the use of broad-spectrum antibiotics in patients with ventilator-associated pneumonia (VAP), which is one of the most common hospital-acquired infections in ICUs.

**Methods/design:**

The GRACE-VAP trial is a multicentre, randomised, open-label parallel-group trial to assess the non-inferiority of Gram stain-guided initial antibiotic treatment to guidelines-based initial antibiotic treatment for the primary endpoint of clinical response rate in patients with VAP. Secondary endpoints include the coverage rates of initial antibiotic therapies, the selected rates of anti-pseudomonal agents and anti-methicillin-resistant *Staphylococcus aureus* (anti-MRSA) agents as initial antibiotic therapies, 28-day all-cause mortality, ICU-free days, ventilator-free days and adverse events. Patients are randomly assigned to receive Gram stain-guided treatment or guidelines-based treatment at a ratio of 1:1. In the Gram stain group, results of Gram staining of endotracheal aspirate are used to guide the selection of antibiotics. In the guidelines group, the combination of an anti-pseudomonal agent and an anti-MRSA agent is administered. A total sample size of 200 was estimated to provide a power of 80% with a one-sided alpha level of 2.5% and a non-inferiority margin of 20%, considering 10% non-evaluable patients.

**Discussion:**

The GRACE-VAP trial is expected to reveal whether Gram staining can reduce the use of broad-spectrum antibiotics without impairing patient outcomes and thereby provide evidence for an antibiotic selection strategy in patients with VAP.

**Trial registration:**

Clinicaltrials.gov, NCT03506113. Registered on 29 March 2018.

University Hospital Medical Information Network, UMIN000031933. Registered on 26 March 2018.

**Electronic supplementary material:**

The online version of this article (10.1186/s13063-018-2971-2) contains supplementary material, which is available to authorized users.

## Background

The increasing prevalence of infections caused by multidrug-resistant (MDR) organisms in intensive care units (ICUs) is recognised as a significant health threat all over the world [1, 2]. However, the development of new pharmaceutical agents is dwindling. Therefore, antibiotic options for the treatment of MDR organisms are becoming increasingly limited [3, 4]. To alleviate this issue, optimising the use of antibiotic agents has been emphasised [5].

Antibiotics are extensively used in ICUs because of the increased risks of infection due to underlying diseases or conditions, impaired immunity, and exposure to multiple invasive devices. In fact, the Extended Prevalence of Infection in Intensive Care II study showed that 51% of patients were considered to be infected during their stay in ICUs [6]. Ventilator-associated pneumonia (VAP) is an important complication that generates a need for antibiotic administration in patients with mechanical ventilation because it is one of the most common hospital-acquired infections in ICUs [6–8]. Therefore, the overuse of broad-spectrum antibiotic agents in patients with VAP could be a major cause of the accelerated emergence of antimicrobial-resistant organisms [9]. The 2016 clinical practice guidelines [10] for VAP published by the Infectious Diseases Society of America (IDSA) and the American Thoracic Society (ATS) recommend that an empirical treatment should include coverage for methicillin-resistant *Staphylococcus aureus* (MRSA) and *Pseudomonas aeruginosa*. Although empirical broad-spectrum treatments help to ensure that infections are treated effectively, overuse of broad-spectrum antibiotic agents leads to an increase in antimicrobial-resistant organisms. Thus, there is an increasing need to establish methods to safely reduce the use of broad-spectrum antibiotic agents for VAP.

In this trial, we will adopt a traditional but credible method, the Gram stain, to estimate pathogens of VAP. Gram staining of respiratory samples is potentially useful to guide appropriate initial antibiotic therapy. Several studies have evaluated the correlation between the results of Gram staining and culture, but the results were conflicting [11–16]. Furthermore, the evidence for Gram stain-guided initial antibiotic treatment for VAP is not well established. Therefore, whether the results of Gram staining are accurate enough to safely restrict the use of broad-spectrum antibiotics is still controversial.

We hypothesise that antibiotic treatment based on Gram stain results can restrict the administration of broad-spectrum antibiotic agents for VAP without detrimental effects on patient outcomes. Thus, we planned the GRam stain-guided Antibiotics ChoicE for VAP (GRACE-VAP) trial to investigate whether Gram stain-guided antibiotic treatment is non-inferior to guidelines-based treatment with respect to the clinical response rate in patients with VAP.

## Methods/design

### Design

We will conduct a multicentre, open-label, randomised, non-inferiority trial with blinded endpoint assessment that will compare the Gram stain-guided treatment with standard antibiotic treatment for patients with VAP. The final trial report will follow the Consolidated Standards of Reporting Trials (CONSORT) statement and its extension to non-inferiority and equivalence trials [17]. This study was registered at clinicaltrials.gov under the registry number NCT03506113. The study protocol was written in accordance with the Standard Protocol Items: Recommendations for Interventional Trials (SPIRIT) guidelines (Additional file 1).

### Setting

The study will be conducted in the ICUs of ten tertiary referral hospitals in Japan (Table 1).

**Table 1:** Participating institutions and investigators

Institution	Investigators
Chukyo Hospital	Akinori Osuka, MD, PhD
Ebina General Hospital	Takeshi Yamagiwa, MD, PhD
Hitachi General Hospital	Kensuke Nakamura, MD, PhD
Kansai Medical University Hospital	Hiroki Takahashi, MD
Kansai Medical University Medical Center	Masahiro Kawada, MD
Nagasaki University Hospital	Shuhei Yamano, MD
Osaka General Medical Center	Jumpei Yoshimura, MD
Saga University Hospital	Hiroyuki Koami, MD, PhD
University of the Ryukyus Hospital	Takayuki Taira, MD
Wakayama Medical University Hospital	Kyohei Miyamoto, MD

### Patients

All patients admitted to the ICU will be screened every day during their ICU stay by the study investigators. The study participants will be enrolled in this study if they meet all of the inclusion criteria and none of the exclusion criteria. Patients will be included if they (1) are aged 15 years or older; (2) have undergone mechanical ventilation for at least 48 h; and (3) are diagnosed as having VAP, which is defined by a modified clinical pulmonary infection score of 5 or more [18].

Patients will be excluded if they (1) have an allergy to study medications, (2) are pregnant, (3) have already been discharged from the ICU, (4) are diagnosed as having heart failure or atelectasis, (5) have been administered antibiotics for more than 24 h when they meet the inclusion criteria, (6) are declined to provide full life support, or (7) are judged as inappropriate for inclusion at the discretion of the study physician.

### Ethics and informed consent

The clinical trial will be carried out according to the principles of the Declaration of Helsinki and Ethical Guidelines for Medical and Health Research Involving Human Subjects published by the Ministry of Health, Labour and Welfare of Japan and the Japanese Ministry of Education, Culture, Sports, Science and Technology. The study protocol was approved by the ethics committees of each participating hospital. Written informed consent will be obtained from all patients or their representatives.

### Randomisation and allocation concealment

Patients are randomly allocated to each treatment arm at a 1:1 ratio no earlier than 1 day before starting antibiotic therapy for VAP. Randomisation will be performed with the use of a stochastic minimisation procedure stratified by study centre, presence of chronic obstructive pulmonary disease, presence of traumatic brain injury, presence of post-cardiopulmonary arrest syndrome, and prior antibiotic therapy during the hospitalisation. We will use an electronic data capture system to conduct randomisation and data collection.

### Trial interventions

**Fig. 1:**
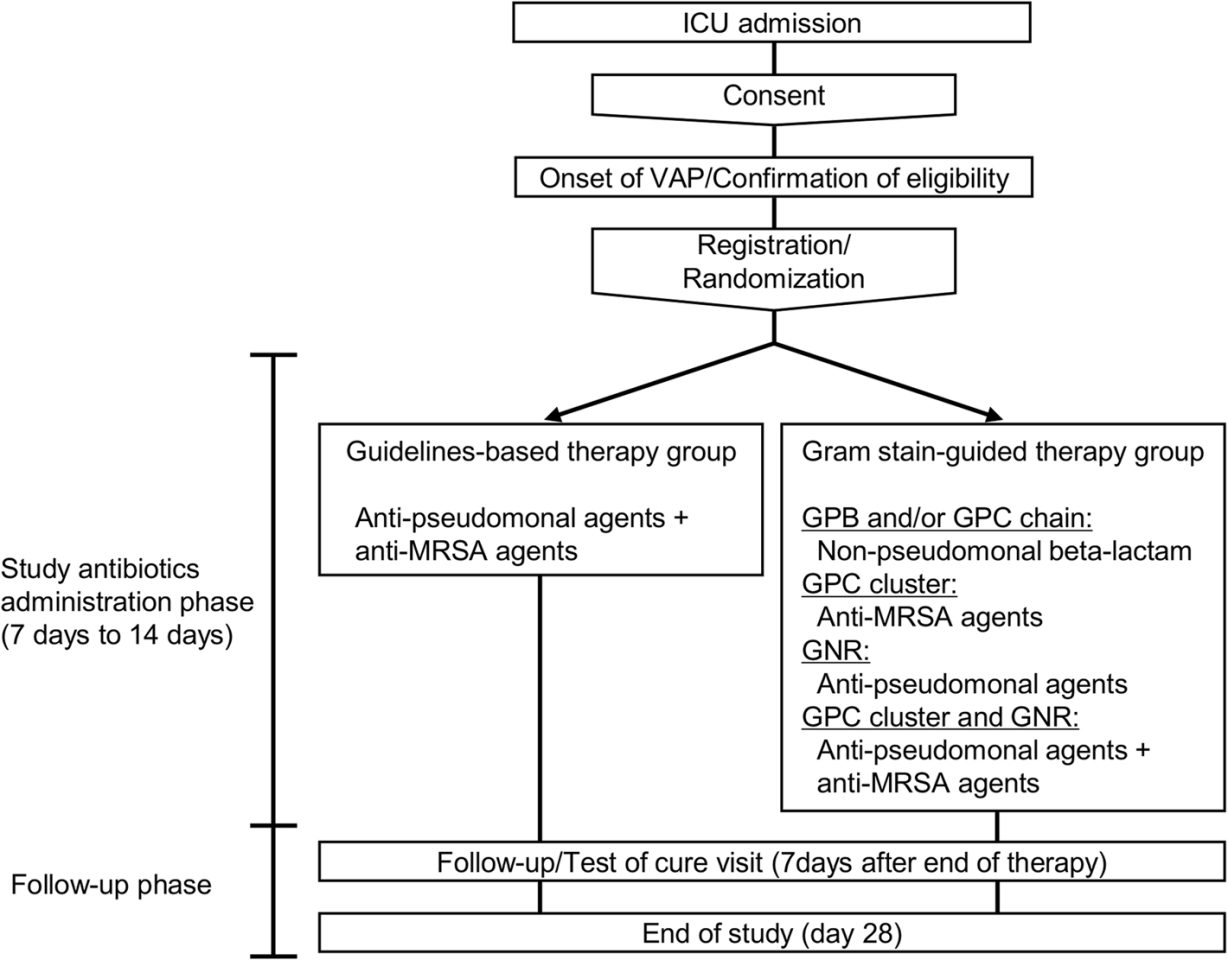
Study design. *GPB* Gram-positive bacilli, *GPC* Gram-positive cocci, *GNR* Gram-negative rods, *ICU* Intensive care unit, *MRSA* Methicillin-resistant *Staphylococcus aureus*, *VAP* Ventilator-associated pneumonia

Treatment of patients in this trial follows a pragmatic approach. All study medications are commercially available and approved in Japan. They are used as marketed in standard dosage regimens. According to their treatment group, patients will receive either antibiotics selected according to the results of Gram staining or standard therapy based on the IDSA/ATS guidelines [10] (Fig. 1).

In the Gram stain group, the results of Gram staining of endotracheal aspirate are used to guide the selection of antibiotics. The results of the Gram stains are categorised as Gram-positive cocci (GPC) chains, GPC clusters, Gram-positive bacilli (GPB), Gram-negative rods (GNR), or a combination of these. A non-pseudomonal β-lactam antibiotic is selected when the Gram stain of the endotracheal aspirate shows only GPC chains and/or GPB. An anti-MRSA agent is selected when the Gram stain results show GPC clusters without GNR. An anti-pseudomonal agent is selected when the Gram stain results show GNR without GPC clusters. The combination of an anti-pseudomonal agent and an anti-MRSA agent is selected when the Gram stain results show both GPC clusters and GNR. We escalate an initial treatment selection process to cover pathogens isolated from respiratory samples before the onset of VAP if the Gram stain results suggest their involvement.

In the standard group, patients are administered the combination of an anti-pseudomonal agent and an anti-MRSA agent according to IDSA/ATS guidelines, because 47.7% of *S. aureus* isolates are MRSA in Japanese ICUs (https://janis.mhlw.go.jp/report/open_report/2016/3/1/ken_Open_Report_201600.pdf). If drug-resistant pathogens are isolated from respiratory samples before the onset of VAP, we escalate an initial treatment selection process to cover them. In both groups, specific antibiotic agents are selected according to previously recorded antimicrobial resistance patterns in each ICU.

The study medication can be de-escalated or escalated to a definitive treatment level according to the results of the pathogens isolated from respiratory samples. Dose adjustments in individual patients are performed as judged appropriate by the site investigator. Study medications are continued for at least 7 days and discontinued on the basis of the site investigator’s judgement.

### Assessment and follow-up

**Fig. 2:**
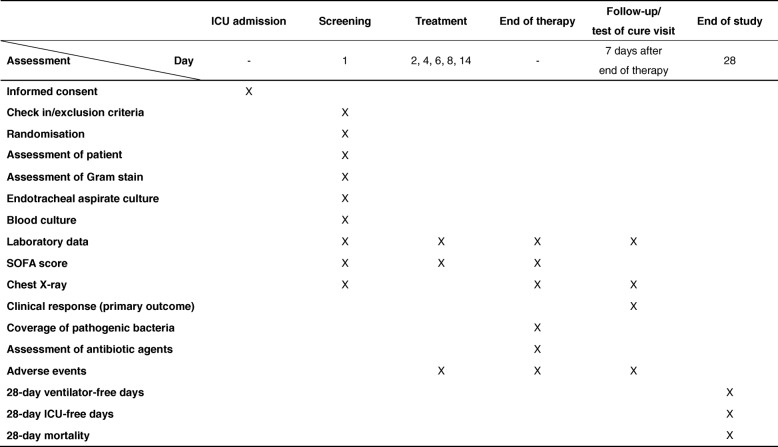
Schedule of assessments. *ICU* intensive care unit, *SOFA* Sequential Organ Failure Assessment

Clinical assessments are performed at baseline and daily throughout the study treatment, at the end of therapy (EOT) and at the follow-up/test of cure (FU/TOC) visit (Fig. 2). Laboratory assessments such as renal function, liver function, platelet count and inflammatory markers including white blood cell count, C-reactive protein and procalcitonin will be performed on the randomisation day; days 2, 4, 6, 8 and 14; and at EOT. Endotracheal aspirates and blood samples for bacterial culture will be obtained on the day of randomisation. We will collect data on all pathogens isolated with at least 1+ semi-quantitative growth from endotracheal aspirates, all pathogens isolated from blood, and antibiotic sensitivity. We will also collect data on all antibiotic agents administered during the VAP treatment, the presence of escalation or de-escalation and the length of the antibiotic therapies. Patients are followed for 28 days to evaluate efficacy and safety variables. If a patient is discharged from hospital prior to 28 days after randomisation, the investigators will contact the patient (or the patient’s representative as appropriate) by telephone to determine the patient’s disposition and status.

### Outcome measures

The primary outcome is the clinical response rate at FU/TOC. Clinical response is defined as that fulfilling all four of the following components: completion of antibiotic therapy within 14 days, improvement or lack of progression of baseline radiographic findings at EOT, and resolution of signs and symptoms of pneumonia at FU/TOC. The signs and symptoms of pneumonia are defined as body temperature ≥ 38 °C, increase in purulent sputum and deterioration of oxygenation. The site investigators will collect information relevant to the clinical response described above. On the basis of these individual data, blinded evaluators on the event adjudication committee will judge the achievement of primary outcome.

The secondary outcomes are the coverage rate of initial antibiotic therapies, the selected rates of anti-pseudomonal agents and anti-MRSA agents as initial antibiotic therapies, 28-day mortality, 28-day ICU-free days, 28-day ventilator-free days, and adverse events. Therapy will be considered appropriate when all pathogens isolated with at least 1+ semi-quantitative growth from endotracheal aspirates are covered by the selected antibiotic agents. Safety outcomes are death during the study period and any adverse events reported at FU/TOC, including renal impairment, thrombocytopenia, diarrhoea, *Clostridium difficile* infection, skin rash and seizure.

### Sample size

The primary efficacy analysis will assess the non-inferiority of the clinical response of Gram stain-guided antibiotic therapy compared with standard therapy. The margin of non-inferiority is set at 20% on the basis of statistically acceptable tolerance and clinically acceptable margin. This margin was used in previous large clinical trials in the field of critical care [19–21]. Then, the non-inferiority of Gram stain-guided antibiotic therapy is concluded if the upper limit of the one-sided 97.5% CI for the difference in clinical response (standard – Gram stain-guided) is less than 20%. To achieve a power of 80% with an α level of 5%, assuming as stated in the previous study that the clinical response rate is 67.8% with standard therapy and is the same with Gram stain-guided antibiotic therapy (unpublished data), with a non-inferiority margin of − 20%, 86 patients are needed in each group. Assuming 10% non-evaluable patients, we decided to enrol 100 patients per group.

### Statistical analysis

Because this is a non-inferiority trial, we will compare the proportion of patients meeting the definition of clinical response using per-protocol analysis as a primary analysis. The per-protocol analysis population will consist of all randomised patients who are not lost to follow-up and have no major protocol deviations. We will also conduct an intention-to-treat analysis as a secondary analysis. We will attest the non-inferiority of the primary outcome on the basis of the normal theory test for binomial proportions. We will conduct the primary analysis without adjustment of potential confounders. We will construct multivariable logistic models or Cox proportional models including the stratified variables as the secondary analysis. We will conduct other post hoc exploratory analyses based on the recommendations of the steering committee. Because of the exploratory nature of these analyses for other than the primary endpoint, no correction for multiplicity is made.

Secondary outcomes will be analysed under a superiority or non-inferiority assumption, as appropriate. Pre-defined subgroup analyses for the primary and secondary endpoints include (1) patients who received prior antibiotic therapy during the hospitalisation versus those who did not, (2) length of ICU stay ≥ 5 days versus < 5 days before randomisation, and (3) septic shock versus no septic shock.

All *P* values are two-sided, and *P* < 0.05 is considered significant other than for the non-inferiority test for clinical response, for which a one-sided *P* < 0.025 is considered significant. All statistical analyses will be performed using JMP 13.1 and SAS 9.4 software (both from SAS Institute Inc., Cary, NC, USA).

### Trial oversight

The trial will be managed by the Division of Trauma and Surgical Critical Care, Osaka General Medical Center, Osaka, Japan. The data centre is located at the Institute for Clinical Effectiveness, Kyoto, Japan, and data managers will centrally monitor the data through the study period. A steering committee was involved in protocol development and will oversee study progress (Table 2). A Data and Safety Monitoring Board (DSMB) made up of independent experts who are not involved in the conduct of the trial will oversee the safety of the trial and efficacy of the trial therapy and will monitor the integrity and validity of the data collected and the conduct of the clinical trial. A scheduled interim analysis will not be performed in this trial. The data centre will report to the steering committee monthly the numbers of registration and mortality as well as occurrence of serious adverse events. When the absolute difference in mortality becomes greater than five patients or any concerns develop, the steering committee will consult the DSMB on the need for an interim analysis. The DSMB will independently perform the interim analysis and make a recommendation to the steering committee whether to continue the trial.

**Table 2:** Study oversight

Role in study	Name	Institution
Principal investigator	Jumpei Yoshimura, MD	Division of Trauma and Surgical Critical Care, Osaka General Medical Center
Steering Committee		
Steering Committee	Kazuma Yamakawa, MD, PhD	Division of Trauma and Surgical Critical Care, Osaka General Medical Center
Steering Committee	Takeshi Morimoto, MD, PhD, MPH	Department of Clinical Epidemiology, Hyogo College of Medicine
Steering Committee	Yoshinori Ohta, MD	Division of General Medicine, Department of Internal Medicine, Hyogo College of Medicine
Event Adjudication Committee	Kazuma Yamakawa, MD, PhD	Division of Trauma and Surgical Critical Care, Osaka General Medical Center
Event Adjudication Committee	Hiroki Takahashi, MD	Kansai Medical University Hospital
Event Adjudication Committee	Kyohei Miyamoto, MD	Wakayama Medical University Hospital
Data and Safety Monitoring Board	Yuichiro Oba, MD, DTM&H	Department of General Internal Medicine and Infectious Diseases, Osaka General Medical Center
Data and Safety Monitoring Board	Atsushi Hiraide, MD, PhD	Critical Care Medical Center, Faculty of Medicine, Kindai University
Data and Safety Monitoring Board	Hitoshi Yamamura, MD, PhD	Department of Critical Care Medicine, Graduate School of Medicine, Hirosaki University
Study statistician	Takeshi Morimoto, MD, PhD, MPH	Department of Clinical Epidemiology, Hyogo College of Medicine
Study secretariat	–	Division of Trauma and Surgical Critical Care, Osaka General Medical Center
Project management	–	Division of Trauma and Surgical Critical Care, Osaka General Medical Center
Data management	–	Institute for Clinical Effectiveness

## Discussion

The GRACE-VAP trial will assess whether initial Gram stain-based restriction of antibiotic therapy is non-inferior to guidelines-based initial broad-spectrum antibiotic therapy in patients with VAP in terms of the clinical response rate. If non-inferiority is achieved, the Gram stain-guided antibiotic choice will be considered to have several advantages in clinical settings. First, Gram staining can be a point-of-care test for selecting antibiotic agents because it should be easy to access at the bedside clinically. Second, it is an inexpensive examination and easy to install in ICUs all over the world, including developing countries. Third, it is easy to learn how to evaluate the results of Gram staining by the method indicated in this trial because we apply merely a simple classification of bacteria.

To the best of our knowledge, this trial will be the first study to answer the clinical question of whether Gram staining can reduce the use of broad-spectrum antibiotics without impairing patient outcomes. We hope that this trial will have a great impact on the establishment of a novel strategy to optimise the use of antibiotic agents safely and restrict the overuse of broad-spectrum antibiotic agents.

## Trial status

The first patient was recruited on 1 April 2018. The last patient is expected to be recruited in March 2021. Osaka General Medical Center provides central trial management and coordination.

## Additional file

Additional file 1: SPIRIT checklist. (DOC 121 kb)

## Abbreviations

ATS: American Thoracic Society
DSMB: Data and Safety Monitoring Committee
EOT: End of therapy
FU/TOC: Follow-up/test of cure visit
GNR: Gram-negative rods
GPB: Gram-positive bacilli
GPC: Gram-positive cocci
GRACE-VAP: GRam stain-guided Antibiotics ChoicE for Ventilator-Associated Pneumonia trial
ICU: Intensive care unit
IDSA: Infectious Diseases Society of America
MDR: Multidrug-resistant
MRSA: Methicillin-resistant *Staphylococcus aureus*
VAP: Ventilator-associated pneumonia
